# Subcutaneous Implantation Assessment of New Calcium-Silicate Based Sealer for Warm Obturation

**DOI:** 10.3390/biomedicines9010024

**Published:** 2021-01-01

**Authors:** João Miguel Santos, Carolina M. Coelho, Diana B. Sequeira, Joana A. Marques, Joana F. Pereira, Vitor Sousa, Paulo J. Palma, Ana C. Santos

**Affiliations:** 1 Institute of Endodontics, Faculty of Medicine, University of Coimbra, 3000-075 Coimbra, Portugal; carolmpc11@hotmail.com (C.M.C.); dianasequeira@fmed.uc.pt (D.B.S.); joanaamarques@uc.pt (J.A.M.); joaninhafpereira@gmail.com (J.F.P.); ppalma@uc.pt (P.J.P.); 2 Center for Innovation and Research in Oral Sciences (CIROS), Faculty of Medicine, University of Coimbra, 3000-075 Coimbra, Portugal; 3 Center for Neuroscience and Cell Biology (CNC), University of Coimbra, 3004-504 Coimbra, Portugal; 4 Institute of Pathological Anatomy, Faculty of Medicine, University of Coimbra, 3000-548 Coimbra, Portugal; vitorsousa.patol@gmail.com; 5 Coimbra Institute for Clinical and Biomedical Research (iCBR) and Institute of Biophysics, Faculty of Medicine, University of Coimbra, 3000-548 Coimbra, Portugal; acsantos@fmed.uc.pt

**Keywords:** biocompatibility, endodontic sealers, subcutaneous implantation, bioactivity potential, calcium silicate-based sealers, endodontics

## Abstract

Calcium silicate-based sealers were recently introduced as a new class of endodontic sealers, with potential further benefits due to their bioactivity. The aim of this study was to evaluate the biocompatibility of two new hydraulic calcium silicate-based sealers, TotalFill BC Sealer (FKG, La Chaux-des-Fonds, Switzerland) and TotalFill BC Sealer HiFlow (FKG, La Chaux-des-Fonds, Switzerland) through subcutaneous implantation in connective tissue of rats. Subcutaneous implantation was performed in 16 young Wistar rats. Four polyethylene tubes were implanted in each animal, one empty to serve as a control, and three filled with tested sealers: AH Plus as reference (Dentsply DeTrey, Konstanz, Germany), TotalFill BC Sealer (BC) and TotalFill BC Sealer HiFlow (HiFlow). Eight rats were euthanized at 8 days and the remaining eight at 30 days. Hematoxylin-eosin staining was used to score the inflammatory reaction, macrophage infiltrate and to measure the thickness of the fibrous capsule. von Kossa staining was performed to evaluate the mineralization level. Kruskal–Wallis test followed by Dunn’s *post hoc* test was used to analyze non-parametric data. To analyze the influence of the implantation time within each material, a Mann–Whitney U test was performed. At eight days post-implantation, AH Plus induced a more intense inflammatory reaction when compared both with the control (*p* ≤ 0.001) and BC (*p* ≤ 0.01). HiFlow presented a higher score of macrophage infiltrate than control (*p* ≤ 0.01) and BC (*p* ≤ 0.05). The fibrous capsule thickness in this period was significantly higher for the BC group when compared to control (*p* ≤ 0.01) and AH Plus (*p* ≤ 0.05). The mineralization potential was higher for the HiFlow group when compared with the control (*p* ≤ 0.001) and AH Plus (*p* ≤ 0.001). At 30 days post-implantation, the score for the inflammatory reaction remained higher for the AH Plus group when compared both to control (*p* ≤ 0.01) and BC (*p* ≤ 0.001). The macrophage infiltrate of the HiFlow was significantly higher than control (*p* ≤ 0.001) and AH Plus groups (*p* ≤ 0.01), additionally, the fibrous capsule of the BC (*p* ≤ 0.001) and HiFlow (*p* ≤ 0.01) groups were both thicker than control. Mineralization potential was observed only on BC (*p* ≤ 0.05) and HiFlow groups (*p* ≤ 0.001), when compared to control). BC exhibited the best biocompatibility performance of all tested sealers and HiFlow provided the greatest induction of mineralized tissues. Both TotalFill BC Sealer and TotalFill BC Sealer HiFlow are biocompatible and show potential bioactivity when implanted in the subcutaneous tissue. Bioactivity was not found in AH Plus.

## 1. Introduction

The purpose of root canal obturation is to prevent microleakage of fluids from the periapical tissues or saliva into the canal as well as bacteria and their by-products and antigens to the periapical tissues [1]. Ideally, the canal should be filled densely to the apical terminus [2]. Clinical studies have shown that flush root fillings (0–2 mm within radiographic apex) were associated with better outcome than short (>2 mm short of radiographic apex) or long (extruded) root fillings [3]. Therefore, to achieve the best prognosis, root filling should be restricted to the intraradicular space [2,3].

Nevertheless, with the current root filling techniques, based upon a gutta-percha core and a root canal sealer, the risk of extrusion into the periradicular tissues is unavoidable. It is present when using cold lateral condensation and it is increased with warm gutta-percha techniques [4]. In a recent clinical study using a single-cone technique associated with a calcium-silicate based sealer, sealer extrusion was observed in 47% of the cases [5]. Extrusion may result from a variety of reasons, including difficulty in establishing a precise limit of canal preparation, patency preparation techniques, apical resorption or via apical foramina and lateral canals [6]. 

The probability of direct and interfacial contact between root canal sealers and periradicular tissues foreground the importance of assessing their biological properties [7]. Their interaction with periradicular cells can slow down the healing process of apical periodontitis [8,9] or act as bioactive stimulators of periapical healing and hard tissue formation [10]. 

Novel sealers based upon calcium silicates were developed and inspired by the excellent sealing ability and biocompatibility of calcium silicate-based cements [10,11]. TotalFill BC Sealer (BC) (FKG, La Chaux-des-Fonds, Switzerland) belongs to this new class of sealers. It is a pre-mixed, ready-to-use, injectable calcium silicate-based sealer, whose main components are calcium silicates, calcium hydroxide, calcium phosphate, thickening agents and zirconium oxide as a radiopacifier agent. Cytotoxicity assays for BC revealed that this sealer demonstrates an excellent biocompatibility with human gingival fibroblasts in vitro [12] and enhanced cell viability, attachment, and mineralization gene expression on human periodontal ligament stem cells [13]. Cytocompatibility and excellent bioactivity of BC were also confirmed on human osteoblast-like cells [14].

However, there are some concerns regarding the BC sealer as it may be affected by temperature, decreasing its flow ability and setting time when heat is applied, which can negatively alter the quality of the obturation when a warm obturation technique is used [15]. Therefore, recently, a specific sealer was developed for this purpose and it is available as TotalFill BC Sealer HiFlow (HiFlow) (FKG, La Chaux-des-Fonds, Switzerland). According to the manufacturer, the physicochemical properties remain stable at temperatures corresponding to those attained inside the root canal during warm procedures. It also provides lower viscosity, lower film thickness and higher flow ability when heated, being more radiopaque than its predecessor [16]. Cell culture cytocompatibility assessment for HiFlow revealed a biocompatibility profile similar to the original BC sealer (BC) [16,17]. 

After showing good in vitro results, biocompatibility evaluation of root canal sealers must include in vivo implantation, in order to have an insight into the subcutaneous connective tissue’s reaction to the materials [18]. The present study is the first research assessing in vivo biocompatibility of HiFlow after two previous in vitro cytotoxicity assays demonstrated good biological properties and potential to promote hard tissue formation [16,17].

The purpose of the current study was to evaluate the biocompatibility and bioactive potential of two new hydraulic calcium silicate-based root canal sealers (BC and HiFlow) compared to a reference sealer, AH Plus (Dentsply DeTrey, Konstanz, Germany), through subcutaneous implantation in a rat experimental model. The null hypothesis was that there would be no difference in histological reactions between groups.

## 2. Materials and Methods

### 2.1. Animal Study

This study followed the ARRIVE (Animal research: Reporting in vivo experiments) guidelines [19]. Sixteen male Wistar rats were used for the in vivo assay (age: 8–10 weeks, body weight: 110–240 g). The sample size was established based upon previous research [18,20]. All procedures were conducted in accordance with the standards of the National Institutes of Health and the Guide for the Care and Use of Laboratory Animals [21]. The Institutional Ethics Committee on the Use of Animals of the Faculty of Medicine of the University of Coimbra approved this study. The animals were observed twice-a-day by the researchers during the whole length of the experiment.

### 2.2. Experimental Protocol

The animals were anesthetized with ketamine 50 mg (Ketalar, Pfizer, Sandwich, United Kingdom) and chlorpromazine 5 mg/mL (Largactil, Laboratórios Vitória, Amadora, Portugal), via intramuscular (i.m.) injections with a dosage of 0.2 mL/100 g in the thigh. Dorsal fur was removed, with the animals positioned in ventral *decubitus*. Incisions were made in each one of the four quadrants of the dorsal region using a No. 15 scalpel blade, equidistant from the spine with an orientation from head to tail. Hence, two scapular and two caudal pockets were created. 

Three polyethylene tubes (9 mm length and 0.9 mm internal diameter) were filled with AH Plus, BC and HiFlow, respectively (Table 1). These sealers were prepared under aseptic conditions and according to the manufacturers’ instructions immediately before implantation. Four polyethylene tubes were implanted subcutaneously per animal. One of the tubes in each animal was left empty being the negative control, and the other three were filled with AH Plus, BC and HiFlow, respectively.

After implantation, the incisions were closed with a non-resorbable silk 3-0 suture (Silkam HR26, B. Braun Surgical, Rubí, Spain). Animals were supervised post-surgery until they were completely awake after anesthesia and were housed in individual boxes. The boxes were packed-up in a ventilated rack system with temperature and air-controlled conditions (Tecniplast, 9ARMI/4120), placed in a dedicated room with light and humidity control. The animals had appropriate food and water *ad libitum*. No adverse events were registered during the immediate post-operative period or during the follow-up until the end of the experiment.

The study comprised two different time periods, 8 and 30 days (*n* = 8 for each time), and four experimental groups. At the end of each experimental period, the animals were sacrificed by anesthetic overdose. The location of the implants was found through tactile sensitivity and surgical removal of the implants was made with adjacent 1 cm safety margins of the surrounding tissues. The biopsy samples were stored in histology cassettes labeled with the animal number, study group and experimental period.

The samples were then fixed in 10% neutral buffered formaldehyde solution (Panreac, Barcelona, Spain) at 4 °C. Samples were prepared for routine histologic procedure. After fixation, each sample was dehydrated in alcoholic solutions of increasing concentrations up to 100%, cleared in xylol, and impregnated and embedded in Paraplast (Paraplast Regular, Sigma Aldrich, St Louis, MO, USA) to form blocks. Finally, these tissue blocks were trimmed and cut into 5 µm sections using a microtome (Leica RM 2155, Leica, Lisbon, Portugal). The thin sections were mounted on glass microscope slides and stained using 2 histologic methods: the routine hematoxylin and eosin (H&E) and the von Kossa (VK) staining protocol in order to evaluate the mineralization level in the tissue/sealer interface. The VK positive structures were observed under polarized light microscopy for the analysis of birefringent structures.

### 2.3. Histological Analysis

**Figure 1:**
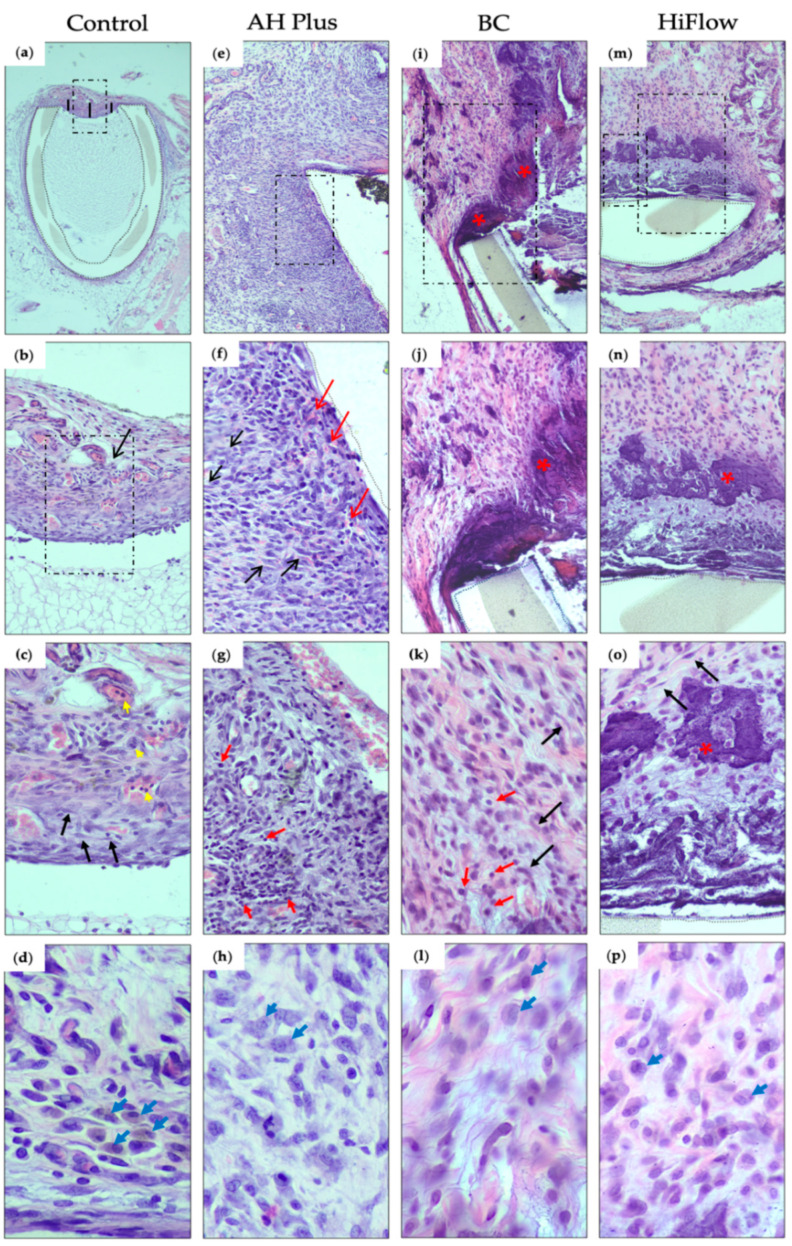
Hematoxylin and eosin histological sections of the interface tissue-sealer 8 days after subcutaneous implantation (dashed boxes mark the view of the subsequent image): (**a**) control group, evidencing the thin fibrous capsule at the interface between the host tissue and the polyethylene tube (demarcation with dotted lines), measured in 3 points (represented by the black lines) (40× magnification); (**b**) the fibrous capsule and mild inflammatory reaction (score 1) (black arrow) at the interface tissue-sealer (200×); (**c**) high magnification showing in detail the cellular population consisting of polymorphonuclear leukocytes (neutrophils) (yellow arrows) and fibroblasts (black arrows) (400×); (**d**) macrophages (blue arrows) (800×); (**e**) AH Plus group, showing granulation tissue surrounding the polyethylene tube (demarcation with dotted lines) (100×); (**f**) high magnification evidencing small congested neo-capillaries (red arrows), fibroblasts (black arrows) and moderate inflammatory reaction (score 2) (200×); (**g**) high magnification, showing the inflammation with mainly lymphocytes (red arrows) and neutrophils (400× hematoxylin and eosin (H&E)). (**h**) Macrophage infiltration (blue arrows) (800×); (**i**) TotalFill BC Sealer (BC) group, showing a fibrous capsule with calcification (bluish deposits) (red asterisk) (100×). (**j**) Higher magnification showing fibroblasts and some inflammatory cells (score 1) and the calcified area in more detail (red asterisk) (200×). (**k**) Fibroblasts (fusiform cells) (black arrows) in a stroma with some collagen fibrils and some lymphocytes (red arrow), plasma cells and rare neutrophils (400×). (**l**) Macrophage infiltration (blue arrows) (800×); (**m**) HiFlow group, revealing a fibrous capsule with calcification next to the polyethylene tube (100×); (**n**) higher magnification to observe fibroblasts, and inflammatory cells (score 1) next to the calcified area (red asterisk) (200×); (**o**) calcified area (red asterisk), with fibroblasts (fusiform cells) (black arrows) in an edematous and low collagenous stroma with lymphocytes (400×); (**p**) macrophage infiltration (800×). (*n* = 8).

The stained histological sections were analyzed with a light microscope (Nikon Eclipse Ci-L, Tokyo, Japan) and digital photos were obtained using an accoupled camera to the microscope (Nikon Digital Sight DS-Fi1, Tokyo, Japan) and analyzed by a blind investigator to the implantation time and sealer. Histological assessment was performed in all biopsy samples; in each group, 5 hematoxylin-eosin and von Kossa-stained sections of the polyethylene tube opening were observed. The most centered section of each sample was selected for further evaluation. Tissue reaction was assessed according to a scoring system (Table 2) for inflammatory reaction in/around the interface sealer/tissue (magnification field 100×), macrophage infiltrate (magnification field 100×), thickness of the fibrous capsule (magnification field 40×: measured with the image software Nikon DS-L3 in 3 points (2 measurements near each of the margins of the top opening of the polyethylene tube and a third one in the middle, according to the example on Figure 1a)) and mineralization (extension of mineralization area on top opening of the tube, observed with magnification field 40×).

### 2.4. Statistical Analysis

Statistical analysis was performed using PRISM8 software (version 8.4.2, GraphPad Software, San Diego, CA, USA). To analyze non-parametric data, a Kruskal–Wallis test was used followed by the Dunn’s *post hoc* test. To analyze the influence of the time within the material, a Mann–Whitney U test was used. The *p*-value for significance was set at 0.05.

## 3. Results

### 3.1. Control Group

**Figure 2:**
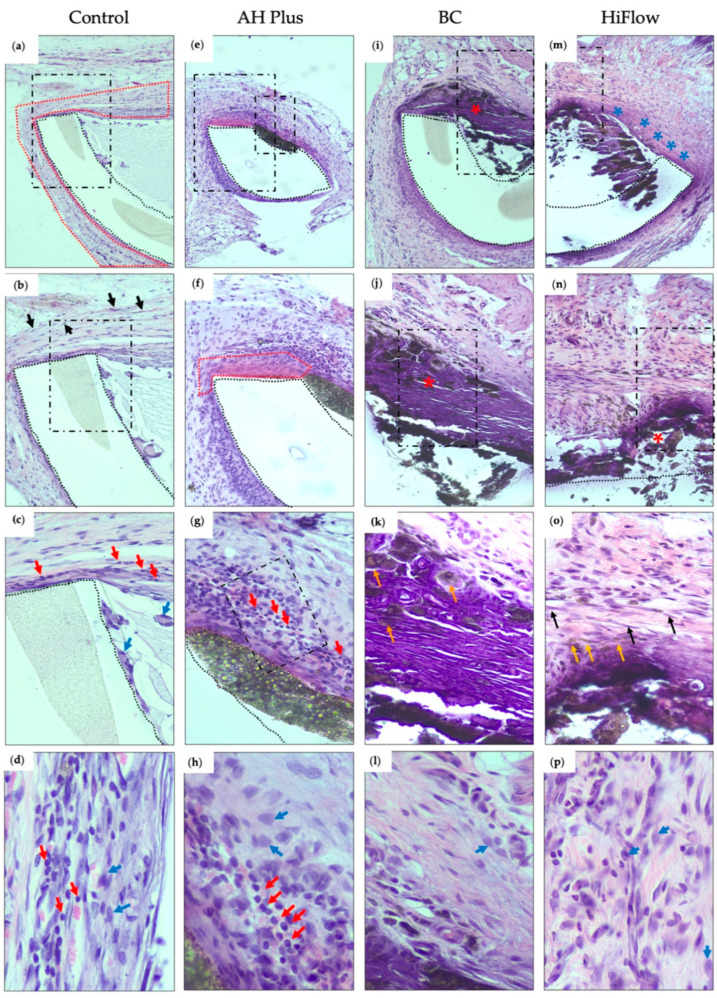
Hematoxylin and eosin histological sections of the interface tissue-sealer 30 days after subcutaneous implantation (dashed boxes mark the view of the subsequent image): (**a**) control group, showing the polyethylene tube (demarcation with dotted lines) and content surrounded by a thin fibrous capsule (demarcation with dotted red line) (100×); (**b**) high magnification detailing the fibrous capsule with fibroblasts (black arrows) and some inflammatory cells (score 1) (200×); (**c**) high magnification showing fibroblasts in collagenous stroma and some lymphocytes (red arrows) and macrophages (blue arrows) adjacent to the polyethylene tube (400×); (**d**) inflammatory infiltrate (800×); (**e**) AH Plus group, showing fibro-inflammatory reaction around the polyethylene space and the material (100×); (**f**) higher magnification demonstrating a thin fibrous capsule (demarcation with dotted red line) and inflammatory cells (score 2) next to the polyethylene tube and the material (200×); (**g**) higher magnification to observe a thin bundle of fibroblasts surrounded and permeated by lymphocytes (red arrows) (400×); (**h**) macrophage (blue arrows) and lymphocytes (red arrow) infiltrate (800×); (**i**) BC group, revealing a thick fibrous capsule with extensive calcification (red asterisk) (100×); (**j**) higher magnification showing intense calcification (bluish aspect) in the fibrous capsule, and the sealer immediately adjacent to the calcified capsule (red asterisk) (200×); (**k**) higher magnification demonstrating birefringent material deposited in the fibrous and calcified capsule (orange arrow) (400× H&E). (**l**) Macrophage infiltrate (blue arrow) (800×); (**m**) HiFlow group, revealing the material inside the polyethylene tube, surrounded by a fibrous capsule (blue asterisk) and calcification of almost the entire capsule opening (100×); (**n**) high magnification with fragmentation of the calcified area/tissue (red asterisk) (200×); (**o**) high magnification revealing in detail the fibroblasts (fusiform cells—black arrow), collagen deposits and calcified deposits inside the fibrous capsule (orange arrow) (400×); (**p**) macrophage infiltration (blue arrow) (800×). (*n* = 8).

At 8 days, a mild inflammatory reaction was observed in 75% of the samples (Table 3) and a thin immature fibrous capsule was present (Figure 1a–c). Some granulation tissue emerged inside the tube. Very few macrophages were observed in both time periods (Figure 1d and Figure 2d). 

**Figure 3:**
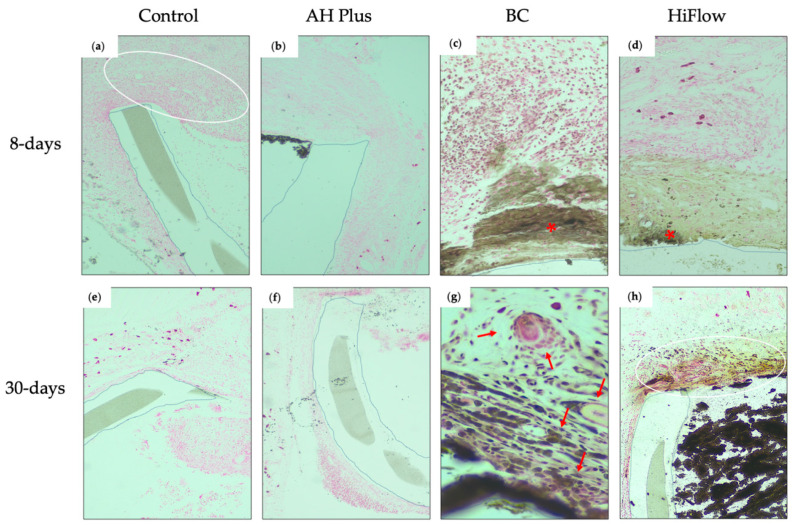
Histological images of the interface tissue-sealer (polyethylene tube demarcation with dotted lines) 8 and 30 days after subcutaneous implantation (von Kossa staining, magnification 200×). Eight days: (**a**) control group, fibrous capsule with fibroblasts and some inflammatory cells (score 1) without mineralization (white oval); (**b**) AH Plus, absence of mineralization around material and next to the fibro-inflammatory capsule; (**c**) BC group, area of mineralization in the capsule (brownish area) and von Kossa positive structures (red asterisk) surrounded by lymphocytes and some neutrophils; (**d**) HiFlow group, fibrous area with mild mineralization (right brownish area) and von Kossa positive structures (red asterisk) in the fibro-inflammatory capsule. Thirty days: (**e**) control group, absence of mineralization; (**f**) AH Pus, thin fibrous capsule without mineralization; (**g**) BC group, birefringent material and mild mineralization in between fibroblasts and surrounded by some inflammatory cells such as lymphocytes, plasma cells, rare neutrophils, and edema (red arrow); (**h**) HiFlow group, moderate mineralization (brownish aspect) in the thin fibrous capsule (white circle) and the sealer (black) inside the polyethylene tube. (*n* = 8 for 8 days; *n* = 8 for 30 days).

After 30 days, the capsule remained thin but surrounded by mature connective tissue and the results showed a resolution of the inflammatory reaction (Figure 2a–c). Mineralization was absent in both periods (Figure 3a,e).

### 3.2. AH Plus

AH Plus showed the highest score for inflammation in both time periods. Images from 8 days showed a moderate inflammatory reaction in 75% of the samples (Figure 1e–g) and a reduced layer of fibrous tissue, forming a thin fibrous capsule in the tissue-sealer interface. The macrophage infiltrate was scored one in half of the samples (Table 3). No mineralization occurred in both time periods (Figure 3b,f). Statistically significant differences were found between groups (*H_(_*_3)_ = 18.20, *p* = 0.0004), in particular, when comparing AH Plus with the control group regarding inflammatory reaction (*z* = 15.63, *n*1 = *n*2 = 8, *p* < 0.001) and with the BC group (*z* = 14.25, *n*1 = *n*2 = 8, *p* < 0.01). At 30 days, a thin fibrocellular capsule (Figure 2e–g) was present and a mild to moderate inflammatory reaction was observed with the presence of lymphocytes and plasma cells. At the 30 day timepoint, there were significant differences between the groups (*H_(_*_3)_ = 17.36, *p* = 0.0004) and AH Plus continued to present a higher inflammatory reaction when compared to control (*z* = 14.25, *n*1 = *n*2 = 8, *p* < 0.01) and BC (*z* = 16.00, *n*1 = *n*2 = 8, *p* < 0.001).

### 3.3. TotalFill BC Sealer

At 8 days, a mild inflammatory reaction was observed, and the number of macrophages was reduced (Figure 1i–k). In the majority of the samples, less than half of the length of the tube opening presented as mineralized (Figure 3c).

Thirty days after implantation there were statistical differences between groups (*H_(_*_3)_ = 19.19, *p* = 0.0002), and TotalFill BC presented the thickest fibrous capsule (Figure 2i–k) and the difference was statistically significant between BC and the control (*z* = 18.25, *n*1 = *n*2 = 8, *p* < 0.001). The macrophage infiltrate (Figure 2l) was scored as one in 62.5% of the samples (Table 3). In the majority of the specimens, mineralization was observed in less than half of the tube opening (Figure 3g).

### 3.4. TotalFill BC Sealer HiFlow

**Figure 4:**
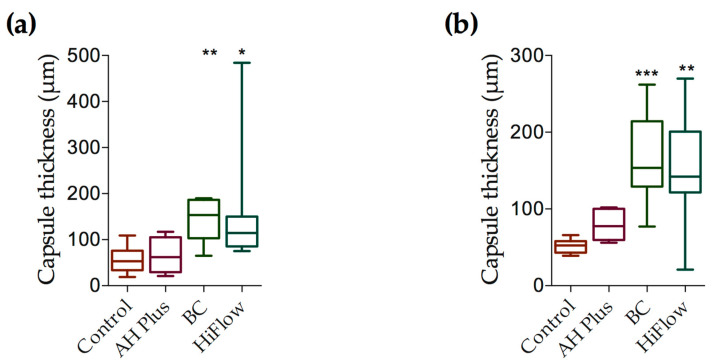
Fibrous capsule thickness (μm) at 8 and 30 days. (**a**) Fibrous capsule at 8 days comparing control to AH Plus, BC and HiFlow groups. Control *n* = 8; AH Plus *n* = 8; BC *n* = 8; HiFlow *n* = 8 (**b**) Fibrous capsule thickness at 30 days. Control *n* = 8; AH Plus *n* = 8; BC *n* = 8; HiFlow *n* = 8. Kruskal–Wallis test with Dunn’s *post hoc* test for multiple comparisons * *p* ≤ 0.05, ** *p* ≤ 0.01, *** *p* ≤ 0.001. Data are presented as means ± SEM.

At 8 days, a mild to moderate inflammatory reaction was present (Figure 1m–o) and statistically different between groups (*H_(_*_3)_ = 13.74, *p* = 0.0033). The macrophage infiltrate (Figure 1p) was significantly higher when compared to control (*z* = 13.25, *n*1 = *n*2 = 8, *p* < 0.001) or BC (*z* = 11.50, *n*1 = *n*2 = 8, *p* < 0.05), and it was considered mild to severe and the highest for this period. The fibrous capsule was different between groups (Figure 4) (*H*_(3)_ = 16.28, *p* = 0.0010) and was thicker than control (*z* = 12.81, *n*1 = *n*2 = 8, *p* < 0.05). Mineralization occurred in all samples (Figure 3d), but to different degrees (*H*_(3)_ = 22.55, *p* < 0.0001), with HiFlow showing highest mineralization potential when compared to control (*z* = 16.13, *n*1 = *n*2 = 8, *p* < 0.001) and AH Plus (*z* = 16.13, *n*1 = *n*2 = 8, *p* < 0.001).

At 30 days, chronic inflammatory reaction was absent or mild (Figure 2m–o) and most of the samples showed a moderate macrophage infiltrate (Figure 2p), which was considered the highest among all groups (*H_(_*_3)_ = 18.76, *p* = 0.0003), with the differences being statistically significant regarding the control (*z* = 16.88, *n*1 = *n*2 = 8, *p* < 0.001) and AH Plus (*z* = 13.13, *n*1 = *n*2 = 8, *p* < 0.01). Mineralization was present in all samples (Figure 3h and Table 3). HiFlow presented the highest mineralization scores amongst all studied groups (*H_(_*_3)_ = 18.76, *p* = 0.0003; *z*_(_HiFlow x control___)_ = 16.36, *n*1 = *n*2 = 8, *p* < 0.001).

### 3.5. Time Comparison

**Figure 5:**
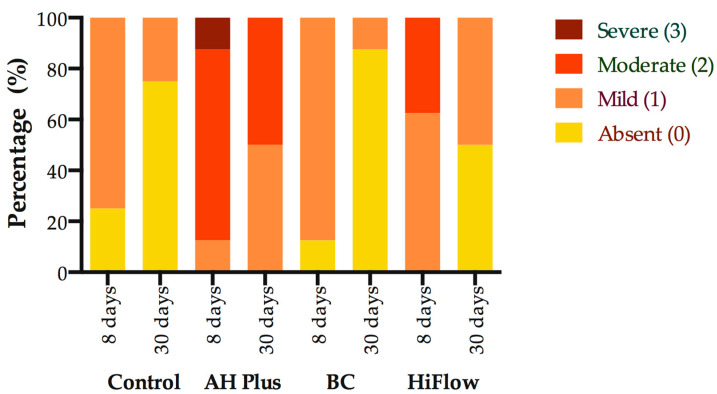
Comparison of inflammatory reaction scores at 8 and 30 days for the control group and root canal sealers. Mann–Whitney U test for within group comparison, *p* ≤ 0.05. Data are presented as percentage of total. (*n* = 8 for 8 days; *n* = 8 for 30 days).

A statistically significant effect on the inflammatory reaction when comparing between 8 and 30 days (Figure 5) was found, with statistical difference for the BC (*U* = 8, *p* = 0.0101) and HiFlow (*U* = 10, *p* = 0.0196) groups, the decrease in inflammatory reaction for BC was Mdn_8_ = 1 to Mdn_30_= 0.0 and for HiFlow Mdn_8_ = 1 to Mdn_30_ = 0.5. Nevertheless, while BC and HiFlow were statistically significant, the inflammatory reaction scores across all experimental groups trended towards a decrease from 8 to 30 days.

Fibrous capsule thickness around tube opening/sealer was higher for BC and HiFlow, compared to control and AH Plus groups, at both time periods (Figure 4). An increase in thickness from 8 to 30 days was observed for HiFlow and BC groups.

## 4. Discussion

In vitro cytotoxicity assays have some limitations and therefore in vivo studies are required to investigate the complex cellular and molecular events involved in the immunoinflammatory response induced by endodontic sealers, which may help tissue repair or sustain chronic inflammatory reaction [22,23]. Subcutaneous implantation within the connective tissue in a rat model is one of the most adequate tests for determining the type and development of local reactions induced by experimental materials [18,20,22]. 

To the best of our knowledge, this is the first in vivo study evaluating TotalFill BC Sealer HiFlow biocompatibility in subcutaneous tissue. The present study shows that BC and HiFlow induced lower inflammatory response when compared to AH Plus, rejecting the null hypothesis and confirming that calcium-silicate based sealers present proper biological properties [16,17]. This arises from the basic composition of the sealers and may also be related to the presence of zirconium oxide in BC, which has been associated with lower inflammatory reaction compared to sealers with barium oxide in their composition [24,25]. 

The inflammatory reaction present in the first time period (8 days) may be caused by surgical trauma during the placement of the polyethylene tube into the pouch and also due to eventual toxic effects of the implanted sealers [18,22]. Additionally, when using calcium silicate-based cements, it is normal for an initial inflammatory reaction to occur due to the high alkalinity of these materials, with inflammatory cell recruitment leading to the production and liberation of proinflammatory cytokines [23]. The results of the inflammatory reaction in the AH Plus group were significantly higher than for the other groups, which might be due to the toxicity exhibited when it is freshly mixed, which reduces after setting [18,26]. 

Nevertheless, the severity of the inflammatory reaction decreased after 30 days, not only for the calcium silicate-based sealers but also for AH Plus. These results are in accordance with previous studies that have demonstrated that after setting, AH Plus was no longer cytotoxic to fibroblasts [12] and osteoclastic stimulatory effects of its extracts were dose- and time-dependent [27].

Calcium silicate-based sealers showed, in vitro, potential to stimulate osteoblastic differentiation and to promote overexpression of osteo/cementogenic genes [13,17]. Histologically, this capacity is evaluated by the von Kossa staining technique, which allows for the detection of calcium precipitates, in order to assess the bioactivity of these materials [25]. Both BC and HiFlow demonstrated the ability to induce mineral deposition shortly after implantation. This may be explained by the alkalinity of the medium induced by the calcium ions release, therefore stimulating the formation of hydroxyl apatite and the release of bone morphogenic protein 2 and alkaline phosphatase, and thus contributing to the mineralization process [23]. In order to identify amorphous calcite deposits, the von Kossa birefringence technique was performed and both calcium silicate-based sealers presented irregular structures with calcium deposits in the adjacent capsule at 8 days. AH Plus did not show von Kossa positive structures. The calcium silicate-based sealers showed birefringent mineralized structures in both periods (8 and 30 days).

The present results are in line with previous reports regarding human dental pulp stem cells exposed to EndoSequence BC Sealer showing a significant increase in calcium nodule formation when compared to AH Plus [26]. This may be related to higher calcium ion release by calcium silicate-based sealers [28]. Calcium ions released during setting interact with carbon dioxide in the tissues and originate deposits that are birefringent under polarized illumination [25,29]. Therefore, the results obtained in the present study are in accordance with the referred previous studies regarding the mineralization potential as both calcium silicate-based sealers evidence von Kossa positive structures at both observation periods. However, distinctively from Alves Silva et al. [29], in the present study, the presence of von Kossa positive structures in association with AH Plus was not observed.

The presence of macrophages demonstrates the organism’s attempt to eliminate the foreign material through phagocytosis [23] and to clear necrotic tissues formed due to previous tissue injury [30]. AH Plus showed a decrease in the number of macrophages between both time periods and the HiFlow group was associated with higher macrophage infiltrate in the longer observation period. The maintenance of a high number of macrophages in BC and HiFlow at the longer period may be associated with higher solubility [31] promoting the release of substances and the formation of calcific precipitates [24]. Although it is widely accepted that sealers should preferably stay within the root canal system, the prolonged release of sealer-derived bioactive molecules through apical foramina, lateral canals, or other portals of communication to the periradicular tissues could play an important role in promoting osteoblastic function and hard-tissue healing [32]. Due to the hydrophilic properties of BC, results from in vitro experiments questioned the setting ability of this sealer (do not set after 25 days); however, the assessment of its setting in vivo proved it was achieved after one week [33], highlighting the importance of in vivo hydration conditions on proper setting of hydraulic root canal sealers. 

Indeed, the thickness of fibrous capsules of BC and HiFlow increased overtime in the present study. Nevertheless, interpretation of this finding remains speculative and is up for debate. Some authors [25,30,34] have interpreted the amount of fibrous capsule developed around the sealer as inversely related to the biocompatibility and as a sign of inflammation. Other authors [35,36,37] have considered that the deposition of fibrous capsule around the material is an indication of tissue tolerance. Khalil et al. [30] suggested that increase in fibrous capsule thickness may be related to the effects of mast cells on fibroblast proliferation, which is in accordance with our observations. Moreover, the increase in fibrous capsule thickness observed in the present study for BC and HiFlow, from 8 to 30 days observation, occurred concomitantly with a decrease in the inflammatory reaction towards both sealers. Therefore, fibroblastic proliferation may be compatible with progressive resolution of the inflammatory reaction towards calcium silicate-based sealers.

Some limitations have to be considered regarding this animal model. First, the reactions in subcutaneous tissue were studied but, in a clinical environment, the sealers will interact mainly with periodontal ligament cells, which may respond differently. Second, biocompatibility and bioactivity are desired qualities for endodontic sealers; however, the clinical performance of sealers is dependent upon other physicochemical and handling properties, which influence the capacity of the operator to fill the canal with the adequate extension, without voids and to prevent coronal microleakage [3]. 

The similar results obtained with BC and HiFlow indicate that this new formulation maintained good biocompatibility levels, as well as the ability to form mineralized tissue. BC exhibited the best biocompatibility performance of all tested sealers and HiFlow provided the greatest induction of mineralized tissues. Nevertheless, all the sealers showed an adequate biocompatibility profile at the end of the study. Therefore, it may be assumed that under regular clinical conditions, all sealers tested in this study present good biocompatibility.

## 5. Conclusions

This study indicates that both TotalFill BC sealer and TotalFill BC HiFlow are biocompatible and exhibit potential bioactivity as they favor calcium precipitation when implanted in the subcutaneous tissue. 

At the longer evaluation period (30 days), the inflammatory reaction decreased, and all tested sealers presented an adequate biocompatibility profile. 

## Figures and Tables

**Table 1:** Composition of the sealers according to the manufacturers.

Sealer	Manufacturer	Composition	Lot/Exp
AH Plus	Dentsply DeTrey, Konstanz, Germany	Epoxide paste: diepoxide, calcium tungstate, zirconium oxide, aerosil, pigment; Amine paste: 1-adamantane amine, N, N’-dibenzyl-5-oxa-nonandiamin-1,9, TCD-diamine, calcium tungstate, zirconium oxide, aerosil and silicon oil	1810000177 2020-10-31
TotalFill BC Sealer (BC)	FKG, La Chaux-des-Fonds, Switzerland	Zirconium oxide, calcium silicates, calcium phosphate monobasic, calcium hydroxide, filler and thickening agents	(10)18004SP 2020-12-31
TotalFill BC Sealer HiFlow (HiFlow)	FKG, La Chaux-des-Fonds, Switzerland	Zirconium oxide, tricalcium silicate, dicalcium silicate, calcium hydroxide and fillers	(10)1803SPWF 2020-11-30

**Table 2:** Score system used to evaluate histopathologic features of the specimens.

Scores	0	1	2	3
Inflammatory reaction	Absent with few inflammatory cells	Mild with less than 25 cells	Moderate with 25 to 125 cells	Severe with more than 125 cells
Macrophage infiltrate	Less than 10 cells	10 to 30 cells	More than 30 cells	-
Mineralization	Absent	Less than half the mineralized area	More than half the mineralized area	-

**Table 3:** Absolute and relative frequencies for histologic evaluation of the samples according to groups and study periods (* *n* = 8; ** *n* = 8).

		8-Days *				30-Days **			
Scores		Control	AH Plus	BC	HiFlow	Control	AH Plus	BC	HiFlow
Inflammatory reaction	0	2 (25)	0 (0)	1 (12.5)	0 (0)	6 (75)	0 (0)	7 (87.5)	4 (50)
	1	6 (75)	1 (12.5)	7 (87.5)	5 (62.5)	2 (25)	4 (50)	1 (12.5)	4 (50)
	2	0 (0)	6 (75)	0 (0)	3 (37.5)	0 (0)	4 (50)	0 (0)	0 (0)
	3	0 (0)	1 (12.5)	0 (0)	0 (0)	0 (0)	0 (0)	0 (0)	0 (0)
Macrophage infiltrate	0	8 (100)	4 (50)	7 (87.5)	2 (25)	8 (100)	6 (75)	3 (37.5)	0 (0)
	1	0 (0)	4 (50)	1 (12.5)	2 (25)	0 (0)	2 (25)	5 (62.5)	6 (75)
	2	0 (0)	0 (0)	0 (0)	4 (50)	0 (0)	0 (0)	0 (0)	2 (25)
Mineralization	0	8 (100)	8 (100)	3 (37.5)	0 (0)	8 (100)	8 (100)	2 (25)	0 (0)
	1	0 (0)	0 (0)	4 (50)	6 (75)	0 (0)	0 (0)	5 (62.5)	5 (62.5)
	2	0 (0)	0 (0)	1 (12.5)	2 (25)	0 (0)	0 (0)	1 (12.5)	3 (37.5)

## Data Availability

Data available on request by contacting the corresponding author.
